# FOXC1 drives lung cancer metastasis and functions as a target for AI-enabled anti-metastatic drug discovery

**DOI:** 10.3389/fphar.2026.1868681

**Published:** 2026-09-10

**Authors:** Sisi Cao, Siqi Peng, Zijun Huang, Jinlan Deng, Jiayuan Wu, Mei Qin, Jianpeng Nong, Linhong He, Lin Liu, Meichun Qin, Lin Tao, Jing Lai, Wenkai Chen, Changyue Jiang, Jie Yang, Yingxin Li

**Affiliations:** 1 School of Pharmacy, Guangxi Medical University, Nanning, Guangxi, China; 2 School of Basic Medical Sciences, Guangxi Medical University, Nanning, China; 3 The Second Affiliated Hospital of Guangxi Medical University, Nanning, Guangxi, China; 4 The Fourth People’s Hospital of Nanning, Guangxi, China

**Keywords:** epithelial-mesenchymal transition, FOXC1, lung cancer, metastasis, β-catenin

## Abstract

**Background:**

Lung cancer metastasis remains the leading cause of treatment failure and death. Forkhead box C1 (FOXC1) promotes cancer progression, but its definitive mechanism in lung cancer metastasis and druggable potential remain unclear. This study aimed to clarify the mechanism and develop a FOXC1-targeted inhibitor.

**Methods:**

FOXC1 expression was analyzed using the HCMDB database. Metastatic function was evaluated using migration and invasion assays and experimental pulmonary metastasis models. Dual-luciferase reporter assays and ChIP-PCR were used to characterize the FOXC1/β-catenin feedback loop. Candidate FOXC1-binding compounds were generated using the MolProphet AI platform. Binding was evaluated by SPR and MST, and complex stability was assessed by molecular docking and molecular dynamics simulations.

**Results:**

FOXC1 expression was elevated in metastatic lung cancer lesions. FOXC1 directly bound the β-catenin promoter, whereas β-catenin-activated TCF4 reciprocally stimulated FOXC1 transcription, forming a self-reinforcing positive-feedback loop. FOXC1-I4 bound FOXC1, reduced FOXC1-dependent reporter activity, and inhibited lung cancer cell migration, invasion, and early pulmonary colonization without significantly affecting cell viability at the tested concentrations.

**Conclusion:**

This study identifies a FOXC1/β-catenin positive-feedback loop that promotes lung cancer metastasis and identifies FOXC1-I4 as an AI-designed FOXC1-binding lead compound with anti-metastatic activity in the experimental models used.

## 1 Introduction

Lung cancer remains a major cause of cancer-related death worldwide, and metastasis is the main reason for treatment failure and death (Sung et al., 2026). Despite advances in targeted therapy and immunotherapy, the total 5-year survival rate of patients with metastatic non-small cell lung cancer in the immunotherapy era remains approximately 10.7% (Wang et al., 2025; Brunelli et al., 2025; Xu et al., 2025). Therefore, defining the molecular mechanisms that govern lung cancer metastasis and developing new therapeutic strategies remain urgent priorities.

Forkhead box C1 (FOXC1), a member of the forkhead box transcription factor family (Pei et al., 2025; Zhang et al., 2025a), fulfills critical functions in standard physiological development, including the development of ocular, cardiac, skeletal, and hair follicle tissues (Wang et al., 2016; Li B. et al., 2024; Luo et al., 2025; Prabakaran et al., 2025). Accumulating evidence has confirmed that FOXC1 acts as a promoter of tumorigenesis in a variety of cancers, including breast, colorectal, and renal cell carcinomas (Li J. et al., 2024; Li et al., 2025; Rojo et al., 2025). In lung cancer, FOXC1 overexpression has been associated with an unfavorable prognosis (Wang et al., 2026); however, its specific functional role in regulating metastasis, and detailed molecular mechanisms remain insufficiently clarified.

Epithelial-mesenchymal transition (EMT) is a core biological process enabling tumor cells to acquire migratory and invasive capacities, and the Wnt/β-catenin signaling pathway is a key regulator of EMT in cancer (Zhang C. X. et al., 2025). Based on previous research, FOXC1 promotes metastasis by regulating genes associated with EMT; however, the direct interaction between FOXC1 and β-catenin signaling requires further investigation. Conventional small-molecule discovery is often prolonged by the iterative development and optimization of biologically relevant screening assays, hit confirmation, and lead optimization. Artificial intelligence (AI)-assisted drug discovery can accelerate early-stage candidate generation and prioritization (Bae et al., 2025; Zhang K. et al., 2025). This study therefore aimed to define the regulatory role of FOXC1 in lung cancer metastasis and to identify a FOXC1-targeted lead compound using an AI-assisted platform.

## 2 Materials and methods

### 2.1 Cell culture

Human lung cancer cell lines NCI-H1299 and A549 were obtained from the Cell Bank of Type Culture Collection of the Chinese Academy of Sciences. These cell lines have been STR-verified and were negative for *mycoplasma*. Cells in Dulbecco’s Modified Eagle’s Medium (DMEM; Gibco, United States) were supplemented with 10% fetal bovine serum (FBS; Gibco) and 1% penicillin-streptomycin (Gibco), and maintained in a humidified incubator at 37 °C with 5% CO_2_. Based on preliminary expression profiling, A549 cells with relatively high endogenous FOXC1 expression were used for FOXC1 knockdown, whereas NCI-H1299 cells with relatively low endogenous FOXC1 expression were used for FOXC1 overexpression.

### 2.2 Lentiviral packaging and cell infection

FOXC1 short hairpin RNA (shRNA), overexpression plasmids, and empty control vectors (EX001-38657, EX001-20242) were obtained from GeneCopiea. Lentivirus was packaged in 293Ta cells using the Lenti-Pac™ HIV Packaging Kit (LT001-01; GeneCopiea) and DNA-EndoFectin (GeneCopiea) following the manufacturer’s protocol guidelines. Collect viral fluids 48 h after transfection and dilute them with the Lenti-Pac™ Lentivirus Concentration Reagent (LT007; GeneCopiea). NCI-H1299 and A549 cells were infected for 14 h, and then selected using 2 μg/mL puromycin for 72 h. Overexpression and knockdown efficiency of FOXC1 were determined by qRT-PCR and Western blot.

### 2.3 Western blot analysis

Whole-cell lysates were prepared by adding RIPA lysis buffer (Beyotime, Shanghai, China) containing PMSF protease inhibitor (Beyotime). Proteins (30 μg per lane) were separated by 12.5% sodium dodecyl sulfate-polyacrylamide gel electrophoresis and transferred to polyvinylidene difluoride membranes (Millipore, Billerica, MA, United States). Saturate these membranes with 5% non-fat milk for 20 min at room temperature, incubate overnight with primary antibodies at 4 °C, wash three times with Tris-buffered saline, and incubate with secondary antibodies at room temperature for 2 h. Amplification of chemiluminescence was employed to detect and record protein signals.

### 2.4 Quantitative real-time PCR (qRT-PCR)

Total cellular RNA was extracted with NucleoZOL reagent (Macherey-Nagel, Düren, Germany). PrimeScript RT Reagent Kit (Takara, Kusatsu, Japan) was used to make cDNA from 1 µg of total RNA. A Bio-Rad CFX Manager 3.1 system was used for quantitative PCR, and PowerUp SYBR Green Master Mix (Thermo Fisher Scientific) and β-actin (as the internal reference gene) were employed. Relative gene expression values are given by formula 2^(-ΔΔCt)^ method. All primers were provided by GeneCopiea.

### 2.5 Migration and invasion assays

For migration assays, A549 or NCI-H1299 cells in logarithmic growth were suspended in DMEM with 1% FBS, and 1 × 10^5 cells in 0.2 mL were added to the upper chamber of Transwell inserts (8 μm pore size; Corning, United States). Add 500 μL of DMEM +10% FBS to the lower well. After 8 h, non-migrating cells at the top were collected using a cotton swab. Fix 15 migratory cells in 4% paraformaldehyde for 15 min; stain with 0.1% crystal violet for 30 min; and observe under a light microscope (Olympus, Tokyo, Japan), and count in representative fields using identical imaging criteria. For invasion assays, Matrigel (Corning) was diluted to 220 μg/mL in serum-free DMEM, then, 100 μL was added to each upper well and left at 37 °C for 1.5 h to solidify. Cell seeding, incubation, fixation, staining, imaging, and counting were then performed as described for the migration assay. MTT assay was used to determine the viability of cells after exposure to 0, 2, 4 and 8 μM FOXC1-I4 under the same treatment conditions as the Transwell assay.

### 2.6 Dual-luciferase reporter assay and ChIP-PCR

Dual-luciferase reporter assay: Construct FOXC1- or TCF4-binding sites in the presence of a luciferase reporter gene, then co-transfect with the corresponding expression plasmid using Lipofectamine 3000 (Thermo Fisher Scientific). pRL-TK vector (Promega, Madison, WI, United States) served as the internal standard. 48 h after transfection, measure the activity of luciferase enzyme in a Dual-Luciferase Reporter Assay System (Promega). To evaluate the effect of FOXC1-I4 on FOXC1-dependent transcriptional activity, cells carrying the FOXC1-responsive reporter were treated with vehicle or 4 μM FOXC1-I4, and firefly luciferase activity was normalized to Renilla luciferase activity.

ChIP-PCR: Based on the supplier’s instructions, perform Chromatin Immunoprecipitation using the EZ-ChIP Kit (Millipore). Target sites were amplified by PCR, and the results were shown on a 1.5% agarose gel. Detailed procedures were described previously (Cao et al., 2018).

### 2.7 Design of FOXC1-targeted small-molecule inhibitor

The full-length structure of FOXC1 was predicted using AlphaFold (https://alphafold.ebi.ac.uk/). Candidate binding sites were detected utilizing the Pocket Discovery Function within the AI-driven medication design framework MolProphet (Yang K. et al., 2024) (https://www.molprophet.com/), and 100 candidate molecules were generated using its molecular generative module. Candidates were filtered according to Lipinski-related properties and a druggability score of at least 5 and were ranked by predicted activity. FOXC1-I4 was prioritized for experimental characterization. FOXC1-I4 was purchased from SanOmics at a supplier-reported purity of >95%.

### 2.8 Declaration of generative AI and AI-assisted technologies in the manuscript preparation process

MolProphet was used to predict FOXC1 binding pockets and generate and prioritize candidate molecular structures. It was used as a research tool for molecular design and was not used to generate or edit the manuscript text. The authors independently selected candidates for experimental evaluation, performed the experiments, analyzed the data, and revised the paper. All authors fully approve the contents of this work.

### 2.9 *In vivo* metastasis model and bioluminescence imaging

BALB/c nude mice (5 weeks old) were purchased from Beijing Vital River Laboratory Animal Technology Co., Ltd. and kept in a specific pathogen-free environment at the Laboratory Animal Center of Guangxi Medical University. Both sexes were included in equal proportions. Twenty-four animals were randomly divided into four groups (n = 6 per group; three male and three female) for the FOXC1-I4 study. Luciferase-positive A549 cells (1 × 10^6 cells in 100 μL sterile PBS) were infused via the lateral tail vein. FOXC1-I4 administration began 24 h after tumor-cell inoculation. The compound was formulated in 20% (w/v) injection-grade sulfobutyl ether-β-cyclodextrin and 1% (v/v) DMSO in sterile normal saline and was administered intravenously at 2.5, 5, or 10 mg/kg in a volume of 5 mL/kg. Adjust according to the weight of the animal, and a maximum of 200 μL per mouse was used. Every third day for a total of ten times, an administration was carried out. Control mice received an equivalent volume of vehicle through the same route and according to the same schedule. For bioluminescence imaging, mice were anesthetized with pentobarbital sodium (0.5 mg/10 g) and injected with D-luciferin potassium salt (1.5 mg/10 g; Promega) through the tail vein. Images were acquired 20 min later using an IVIS Lumina LT small-animal imaging system (PerkinElmer, United States). A whole-body region of interest was defined for each mouse using identical selection criteria. Living Image software automatically estimated and subtracted the background, after which the pixel signals within the region of interest were integrated and expressed as counts. Identical acquisition and analysis settings were applied to all animals. Because treatment was initiated 24 h after cell inoculation, this experiment was considered an early-intervention experimental pulmonary colonization model rather than a treatment model of established metastases.

### 2.10 SPR and MST assays

SPR: FOXC1 protein was immobilized on a CM5 sensor chip (Cytiva, United States). FOXC1-I4 was serially diluted to concentrations ranging from 0 to 25 μM and injected in ascending order of concentration. Binding and dissociation curves were analyzed using a Biacore T200 molecular interaction analyzer to calculate the equilibrium dissociation constant (KD).

MST: FOXC1 protein was fluorescently labeled by incubation with NT-647 dye (Nanotemper Technologies, Germany) for 30 min. FOXC1-I4 was serially diluted 2-fold (0–25 μM) and mixed with labeled FOXC1. A Monolith NT.115 device (Nanotemper Technologies) was used to record thermophoretic profiles, and KD constants were determined using MO Affinity Analysis Software v2.3.

### 2.11 Molecular docking and molecular dynamics simulations

Molecular docking: The AlphaFold-predicted structure of FOXC1 was retrieved, and FOXC1-I4 was converted to PDBQT format. A 40 Å × 40 Å × 40 Å binding pocket was defined using Autodock Vina 1.2.2 software to calculate binding energy and characterize interaction modes.

Molecular dynamics simulations: Gromacs 2022.3 was used for the simulation, along with Amber99sb-ildn and the Tip3p solvent model. Assembly experienced energy relaxation, NVT thermalisation (100 ps), NPT pressure stabilisation (100 ps) and 100 ns of unstrained sampling. Root mean square displacement (RMSD), root mean square fluctuation (RMSF), hydrogen bonding, solvent-accessible surface area (SASA) and radius of gyration (Rg) are all typical parameters, and binding free energy (ΔGMMGBSA) were analyzed.

### 2.12 Statistical analysis

All experimental results are presented as the mean ± standard deviation (SD) of three or more separate assays. The t-test for students was used to compare two groups, and one-way analysis of variance (ANOVA) followed by Tukey’s post-hoc test was employed to compare multiple groups. The p-value was less than 0.05 (ns: p > 0.05; *: p < 0.05; **: p < 0.01). All of the above calculations were carried out in SPSS 27.0 software (IBM, Armonk, NY, United States).

## 3 Results

### 3.1 FOXC1 promotes lung cancer cell metastasis *in vitro* and *in vivo*

**FIGURE 1:**
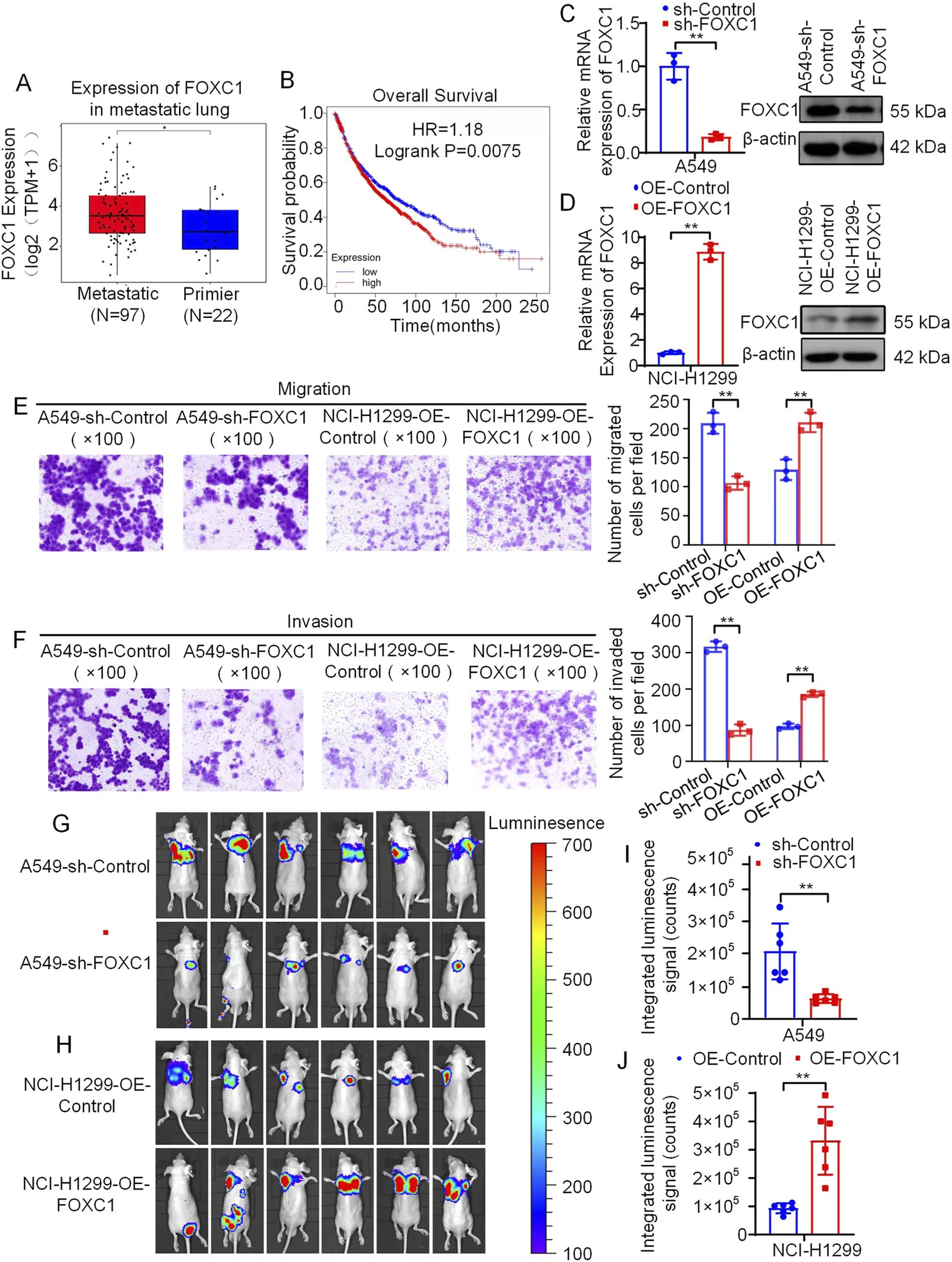
FOXC1 promotes lung cancer cell metastasis *in vitro* and *in vivo*. **(A)** FOXC1 mRNA expression in primary lung cancer tissues and metastatic lesions. **(B)** Kaplan-Meier curves showing overall survival according to FOXC1 expression. **(C,D)** qRT-PCR and Western blot validation of FOXC1 knockdown in A549 cells and FOXC1 overexpression in NCI-H1299 cells. **(E,F)** Representative Transwell migration and invasion images and corresponding quantification of migrated or invaded cells per field (magnification, ×100). **(G,H)** Representative whole-body bioluminescence images from mice inoculated with luciferase-labeled cells. **(I,J)** Quantification of whole-body bioluminescence as integrated luminescence signal (counts) after automatic background correction in Living Image software (n = 6 mice per group). *p < 0.05; **p < 0.01.

Based on the analysis of the HCMDB dataset, FOXC1 expression was relatively high in metastatic lung cancer tissues compared with those from primary lung cancers (Figure 1A). Kaplan-Meier curves drawn by KMplot (https://kmplot.com) show that a high expression of FOXC1 is associated with a shorter overall survival in lung cancer patients (Figure 1B). Based on preliminary findings showing relatively high FOXC1 expression in A549 cells and relatively low expression in NCI-H1299 cells, we established stable FOXC1-silenced A549 cells (A549-sh-FOXC1) and FOXC1-overexpressing NCI-H1299 cells (NCI-H1299-OE-FOXC1). qRT-PCR and Western blotting confirmed the expected changes in FOXC1 mRNA and protein levels (Figures 1C,D). FOXC1 silencing reduced A549 cell migration and invasion, whereas FOXC1 overexpression enhanced these properties in NCI-H1299 cells (Figures 1E,F). Consistent with the *in vitro* findings, the integrated luminescence signal was lower in mice inoculated with A549-sh-FOXC1 cells than in control mice (Figures 1G–I) and was higher in mice inoculated with NCI-H1299-OE-FOXC1 cells than in the corresponding control group (Figures 1H–J).

### 3.2 FOXC1/β-catenin positive feedback loop induces EMT

#### 3.2.1 FOXC1 upregulates β-catenin expression and induces EMT

**FIGURE 2:**
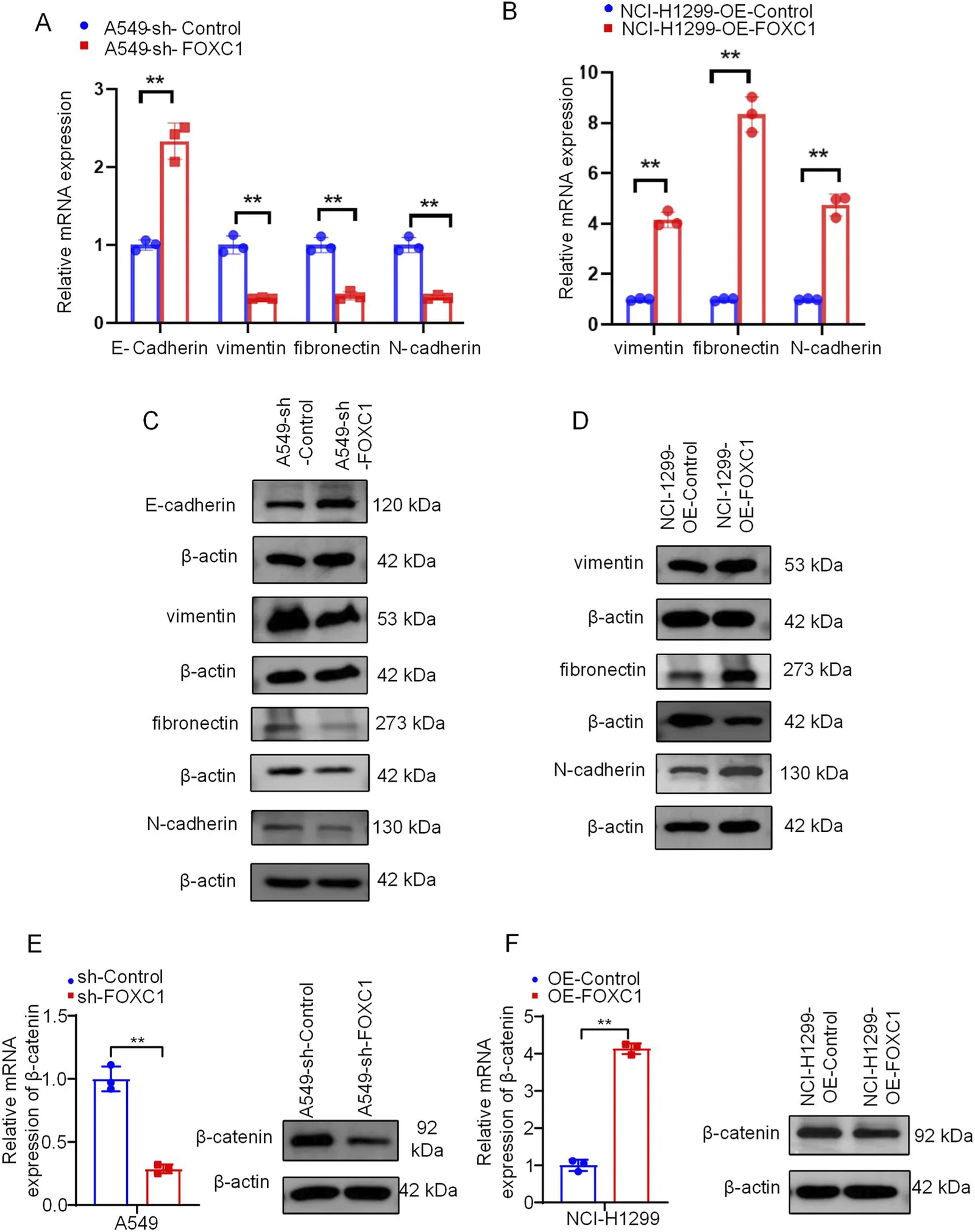
FOXC1 upregulates β-catenin expression and induces EMT. (**A,B)** qRT-PCR analysis of EMT markers in A549-sh-FOXC1 cells and NCI-H1299-OE-FOXC1 cells. **(C,D)** Western blot analysis of E-cadherin, vimentin, fibronectin, and N-cadherin. E-cadherin was below reliable detection in NCI-H1299 cells under the conditions used. **(E,F)** qRT-PCR and Western blot analysis of β-catenin after FOXC1 knockdown or overexpression. **p < 0.01.

Both Western blotting and qRT-PCR results demonstrated that downregulating FOXC1 in A549 cells diminished the mRNA and protein expressions of mesenchymal factors vimentin, fibronectin, and N-cadherin, elevated the epithelial marker E-cadherin (Figures 2A,C), and lowered β-catenin levels (Figure 2E). Conversely, FOXC1 overexpression in NCI-H1299 cells increased mesenchymal-marker expression (Figures 2B,D) and β-catenin expression (Figure 2F). Because NCI-H1299 cells exhibit an endogenous mesenchymal phenotype, E-cadherin was below reliable detection by qRT-PCR and Western blotting under the conditions used. These findings suggest that FOXC1 may promote an EMT phenotype by increasing β-catenin expression.

#### 3.2.2 β-Catenin mediates the pro-metastatic effect of FOXC1 in lung cancer cells

**FIGURE 3:**
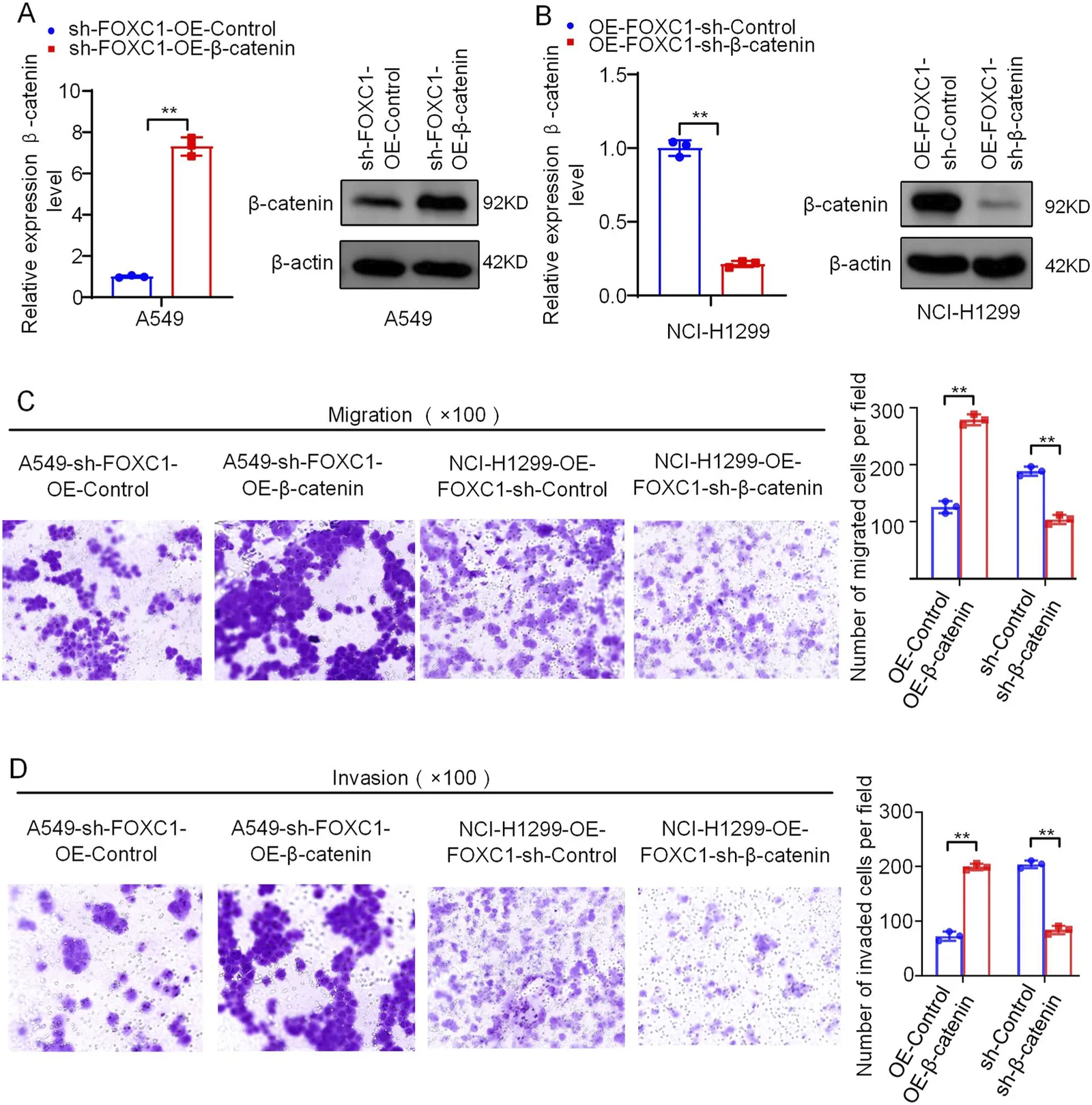
β-Catenin mediates the pro-metastatic effects of FOXC1. **(A,B)** qRT-PCR and Western blot validation of β-catenin overexpression in A549-sh-FOXC1 cells and β-catenin knockdown in NCI-H1299-OE-FOXC1 cells. **(C,D)** Representative Transwell migration and invasion images with corresponding quantification of migrated or invaded cells per field (magnification, ×100). **p < 0.01.

We established β-catenin-overexpressing A549-sh-FOXC1 cells (A549-sh-FOXC1-OE-β-catenin) and β-catenin-silenced NCI-H1299-OE-FOXC1 cells (NCI-H1299-OE-FOXC1-sh-β-catenin) to examine whether β-catenin mediates the pro-metastatic effects of FOXC1. qRT-PCR and Western blotting confirmed β-catenin overexpression and silencing in the respective models (Figures 3A,B). β-Catenin overexpression partially rescued the reduction in migration and invasion caused by silencing FOXC1 in A549 cells. Conversely, β-catenin knockdown attenuated the FOXC1-induced increase in migration and invasion in NCI-H1299 cells (Figures 3C,D).

#### 3.2.3 FOXC1 and β-catenin/TCF4 form a positive feedback loop

**FIGURE 4:**
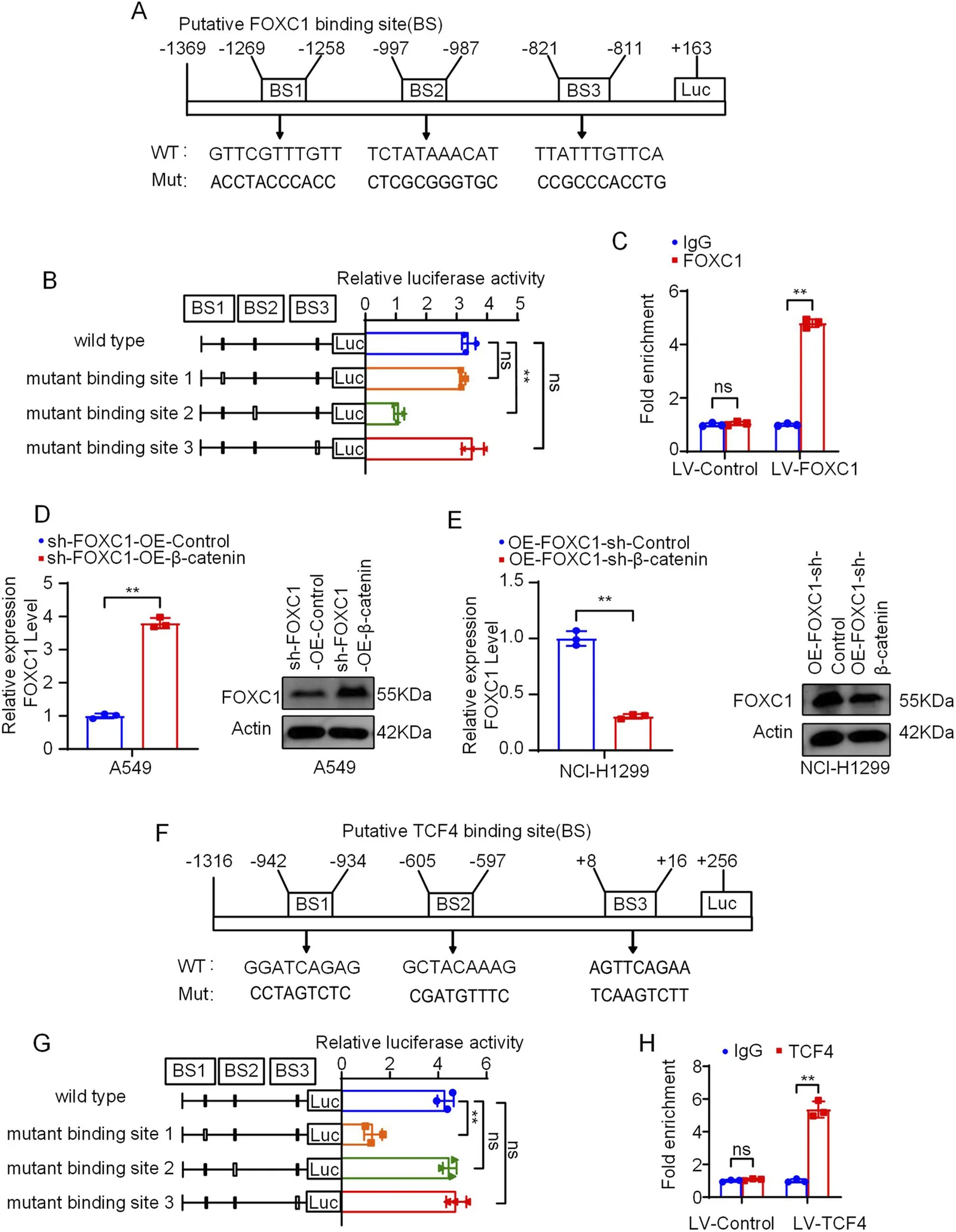
FOXC1 and β-catenin/TCF4 form a positive feedback loop. **(A)** Schematic of putative FOXC1 binding sites (BS1, BS2, BS3) on the β-catenin promoter and mutation sequences. **(B)** Dual-luciferase reporter assay of β-catenin promoter activity with wild-type and mutant FOXC1 binding sites. **(C)** ChIP-PCR analysis of FOXC1 binding to BS2 on the β-catenin promoter. **(D,E)** qRT-PCR and Western blot analysis of FOXC1 overexpression in β-catenin overexpression cells **(D)** and FOXC1 knockdown in β-catenin-knockdown cells **(E)**. **(F)** Schematic of putative TCF4 binding sites (BS1, BS2, BS3) on the FOXC1 promoter and mutation sequences. **(G)** Dual-luciferase reporter assay of FOXC1 promoter activity with wild-type and mutant TCF4 binding sites. **(H)** ChIP-PCR analysis of TCF4 binding to BS1 on the FOXC1 promoter. **p < 0.01.

JASPAR analysis identified three putative FOXC1-binding sites (BS1, BS2, and BS3) in the β-catenin promoter. Mutation of BS2 significantly reduced reporter activity (Figures 4A,B), and ChIP-PCR confirmed FOXC1 enrichment at BS2 (Figure 4C). FOXC1 expression increased after β-catenin overexpression and decreased after β-catenin knockdown (Figures 4D,E). Three putative TCF4-binding sites were also identified in the FOXC1 promoter. Mutation of BS1 markedly reduced reporter activity (Figures 4F,G), and ChIP-PCR confirmed TCF4 enrichment at BS1 (Figure 4H). β-catenin overexpression did not significantly alter TCF4 mRNA or protein abundance (Supplementary Figure S1B), consistent with regulation of TCF4 transcriptional activity rather than TCF4 expression. Together, these data support a self-reinforcing loop in which FOXC1 activates β-catenin transcription and β-catenin-activated TCF4 promotes FOXC1 transcription.

### 3.3 AI-generated FOXC1 inhibitor FOXC1-I4 suppresses lung cancer metastasis *in vitro* and *in vivo*

**FIGURE 5:**
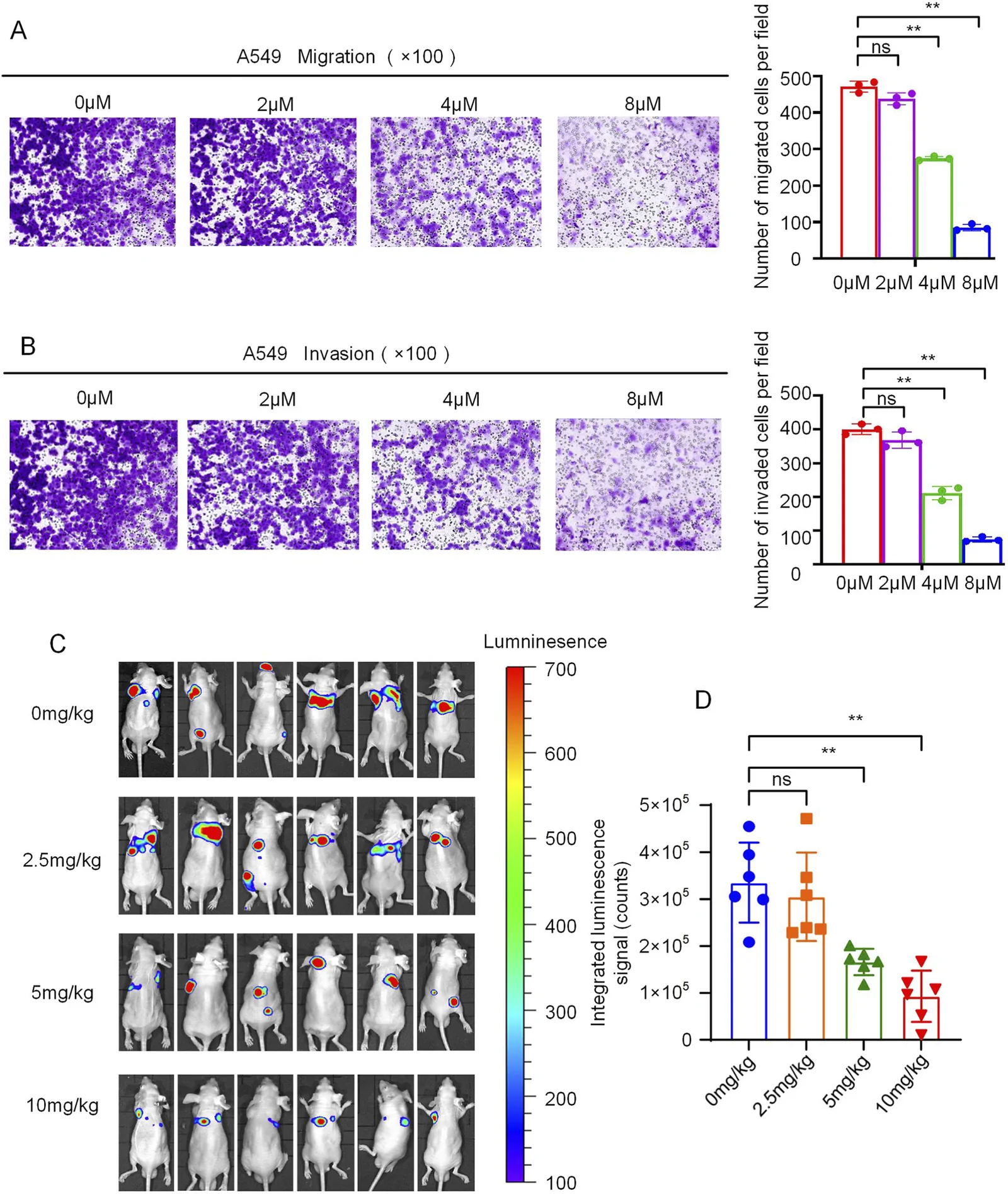
FOXC1-I4 suppresses lung cancer cell migration and invasion *in vitro* and metastasis *in vivo*. (**A,B)** Representative Transwell migration and invasion images of A549 cells treated with FOXC1-I4 at 0, 2, 4, or 8 μM, with quantification of migrated or invaded cells per field (magnification, ×100). **(C)** Representative whole-body bioluminescence images from the early-intervention pulmonary colonization experiment after intravenous FOXC1-I4 treatment at 0, 2.5, 5, or 10 mg/kg (n = 6 mice per group). **(D)** Quantification of whole-body bioluminescence as integrated luminescence signal (counts) after automatic background correction. **p < 0.01.

MolProphet generated 100 candidate FOXC1-targeted molecules (Supplementary Table S1). After druggability and predicted-activity ranking, FOXC1-I4 ((E)-N-(4-hydroxybenzyl)-2-(4-(3-hydroxy-3-phenylprop-1-en-1-yl)phenyl)-N-methylacetamide) was prioritized for experimental characterization; comparative testing of the remaining candidates is ongoing. FOXC1-I4 reduced A549 cell migration and invasion in a concentration-dependent manner at 2, 4, and 8 μM (Figures 5A,B). MTT assays showed no significant reduction in cell viability at these concentrations (Supplementary Figure S1A), indicating that the Transwell effects were not attributable to overt cytotoxicity under the assay conditions. In the early-intervention pulmonary colonization model, FOXC1-I4 reduced the integrated luminescence signal in a dose-dependent manner, with the largest reduction observed at 10 mg/kg (Figures 5C,D). All animals completed the dosing period, and no death or overt deterioration in activity, posture, grooming, or general condition was observed.

### 3.4 Specific binding and mechanism between FOXC1-I4 and FOXC1

**FIGURE 6:**
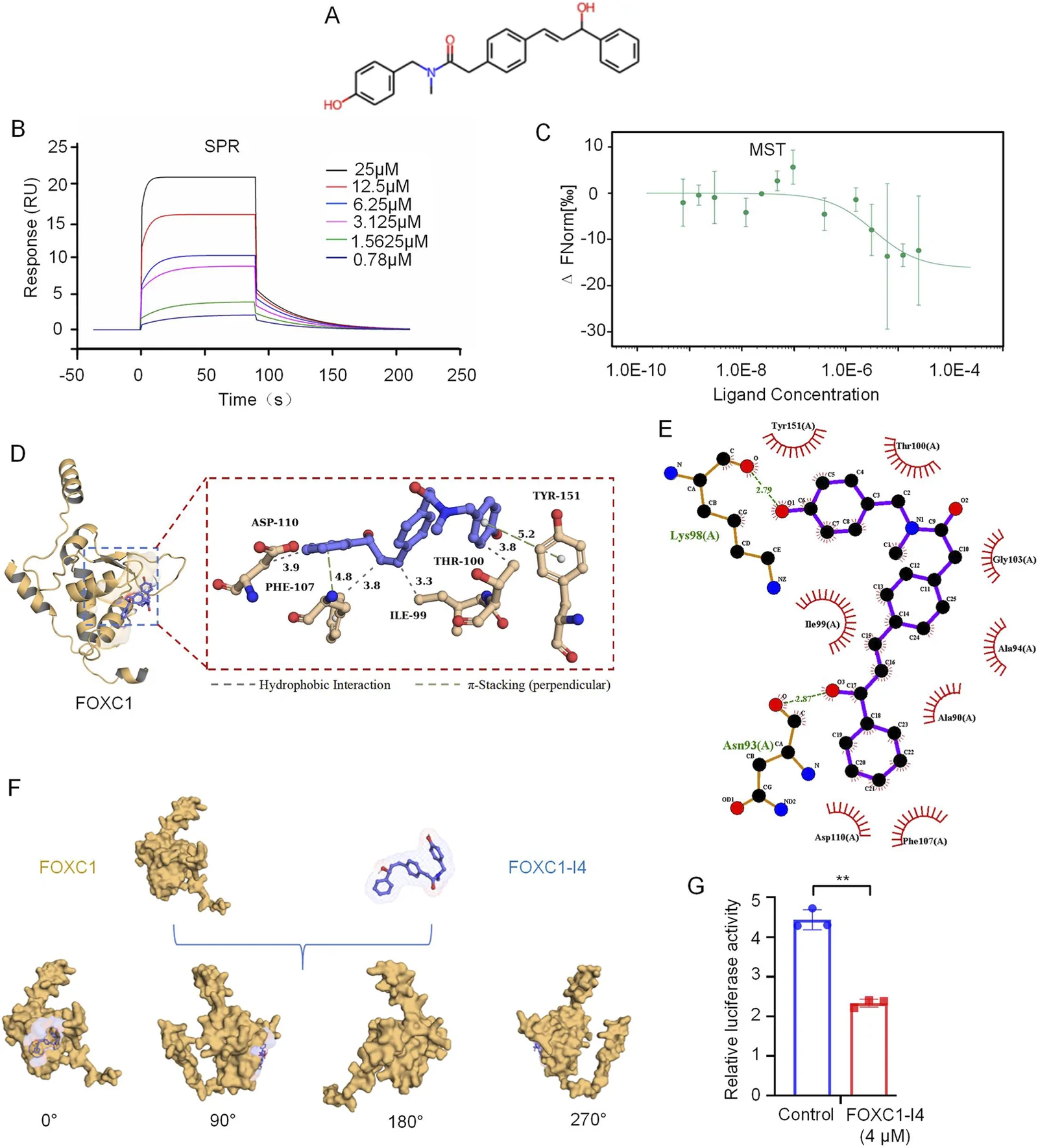
Molecular interaction between FOXC1-I4 and FOXC1 protein. **(A)** Chemical structure of FOXC1-I4. **(B)** Surface plasmon resonance sensorgrams showing concentration-dependent binding of FOXC1-I4 to FOXC1. **(C)** Microscale thermophoresis analysis of FOXC1-I4 binding to FOXC1. **(D)** Three-dimensional docking model and predicted interactions of FOXC1-I4 within the FOXC1 binding pocket. **(E)** Two-dimensional interaction map. **(F)** The same FOXC1-FOXC1-I4 complex viewed at 0°, 90°, 180°, and 270°. **(G)** FOXC1-dependent dual-luciferase reporter activity after treatment with vehicle or 4 μM FOXC1-I4. **p < 0.01.

SPR and MST confirmed direct binding between FOXC1-I4 (Figure 6A) and FOXC1. The SPR-derived equilibrium dissociation constant was 2.68 μM (Figure 6B), and the MST-derived was 3.43 μM (Figure 6C).

Molecular docking calculated a binding energy of −6.6 kcal/mol for the FOXC1-I4-FOXC1 complex. The predicted interaction network included hydrophobic contacts involving Ile99, Thr100, Phe107, and Asp110 and aromatic interactions involving Phe107 and Tyr151 (Figures 6D,E). Figure 6F shows the same FOXC1-FOXC1-I4 complex viewed at 0°, 90°, 180°, and 270°, with FOXC1-I4 located within the predicted binding pocket. In addition, 4 μM FOXC1-I4 significantly reduced FOXC1-dependent luciferase reporter activity compared with vehicle control (Figure 6G), providing functional evidence that FOXC1-I4 suppresses FOXC1 transcriptional activity.

**FIGURE 7:**
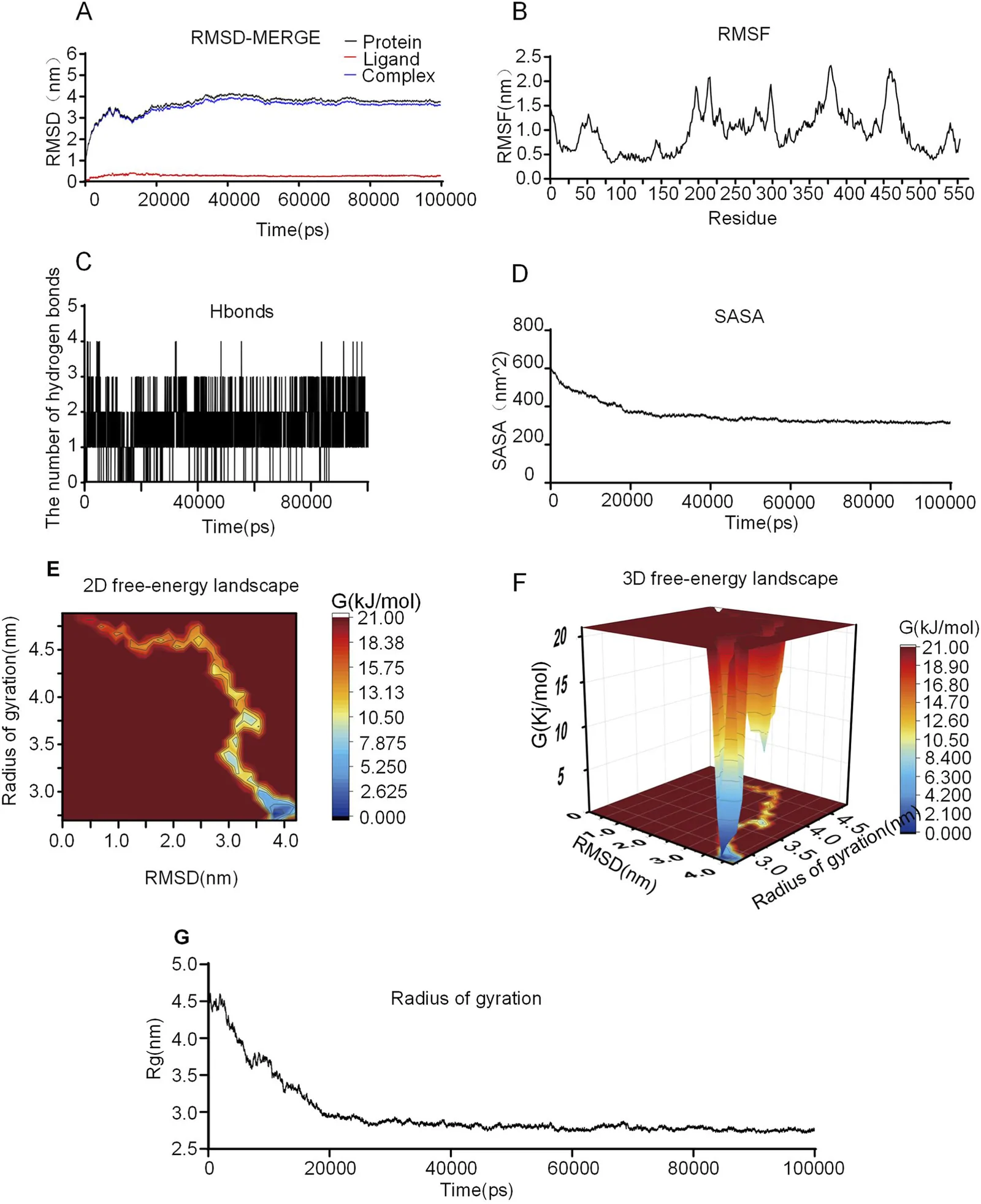
Molecular dynamics simulations of the FOXC1-I4/FOXC1 complex. **(A)** Root mean square deviation (RMSD) of the protein, ligand, and complex during the 100-ns simulation. **(B)** Root mean square fluctuation (RMSF) of FOXC1 residues. **(C)** Number of hydrogen bonds formed between FOXC1-I4 and FOXC1 over time. **(D)** Solvent-accessible surface area of the complex. **(E)** Two-dimensional free-energy landscape plotted against RMSD and radius of gyration. **(F)** Three-dimensional free-energy landscape plotted against RMSD and radius of gyration. The color scales in **(E,F)** represent free energy in kJ/mol. **(G)** Radius of gyration of the FOXC1-FOXC1-I4 complex.

Molecular dynamics simulations showed that the RMSD of the FOXC1-I4-FOXC1 complex reached a plateau during the 100-ns simulation (Figure 7A). RMSF analysis identified relatively limited fluctuations in several regions of the complex (Figure 7B), and one to three hydrogen bonds were maintained during most of the simulation (Figure 7C). The solvent-accessible surface area and radius of gyration decreased and then stabilized, indicating a more compact conformation (Figures 7D–G). The two-dimensional and three-dimensional free-energy landscapes showed a concentrated low-energy basin defined by RMSD and radius of gyration, consistent with a preferred stable conformational state (Figures 7E,F).

## 4 Discussion

This study identifies a FOXC1/β-catenin positive-feedback loop that promotes lung cancer metastasis and characterizes FOXC1-I4 as an AI-designed FOXC1-binding lead compound. The findings extend the mechanistic understanding of FOXC1-driven metastasis and provide preclinical proof-of-concept for pharmacological modulation of FOXC1, while not establishing clinical readiness.

FOXC1 overexpression in lung cancer and its association with poor prognosis are consistent with prior observations (Lin et al., 2017; Yang Y. et al., 2024), underscoring the conserved oncogenic function of FOXC1 across different cancer types.

FOXC1 exerts context-dependent pro-metastatic effects across tumor types. In hepatocellular carcinoma, FOXC1 promotes metastasis through CXCR1/CCL2 activation or DNMT3B-mediated CTH promoter hypermethylation (Huang et al., 2015; Lin et al., 2021); in colorectal cancer, it enhances invasion by upregulating ITGA7 and FGFR4 (Liu et al., 2018); and in breast cancer, FOXC1 drives metastasis through MMP7 induction (Sizemore and Keri, 2012). EMT is a central driver of tumor dissemination, and our findings support a β-catenin-dependent contribution of FOXC1 to EMT-related changes in lung cancer, consistent with the established role of β-catenin in EMT (Maurice and Angers, 2025). The regulatory pattern differs from FOXC1-associated EMT pathways involving ZEB2 in esophageal cancer (Zhu et al., 2017), PI3K-AKT signaling in cervical carcinoma (Huang et al., 2017), and TGF-β/Smad2/3/snail in colorectal cancer (Zhang et al., 2025d), FOXC1 in lung cancer comprehensively modulates EMT by upregulating mesenchymal markers and downregulating epithelial markers via β-catenin. This distinct regulatory pattern aligns with FOXC1’s role in hepatocellular carcinoma microvascular invasion (Xu et al., 2012) but is directly linked to β-catenin for the first time in lung cancer, highlighting a tumor-specific EMT regulatory network.

The direct regulatory interaction between FOXC1 and β-catenin is supported by cross-validation: our team previously demonstrated that FOXC1 promotes stem cell-like characteristics by elevating β-catenin in lung cancer (Cao et al., 2018), and subsequent studies confirmed the FOXC1/β-catenin axis in gastric cancer metastasis (Sun et al., 2022) or synovial fibroblasts proliferation (Wang et al., 2020; Wu et al., 2023). These findings underscore the universal relevance of the FOXC1/β-catenin axis across pathological contexts, reinforcing its potential importance as a therapeutic target.

This study is the first to demonstrate conclusively that in lung cancer cells, FOXC1 directly binds the BS2 site of the β-catenin promoter to promote its transcription, while β-catenin-activated TCF4 binds the BS1 site of the FOXC1 promoter to amplify its expression, forming a self-amplifying positive feedback loop. This bidirectional regulatory mechanism differs from the unidirectional regulation of β-catenin, clarifying the persistent upregulation of FOXC1 and β-catenin in metastatic lung cancer.

FOXC1 expression is regulated at multiple levels. Non-coding RNAs such as circ0061052 (Ma et al., 2020), FOXCUT (Zhang et al., 2020), and circ_0000790 (Yang et al., 2020) modulate FOXC1 through competing endogenous RNA mechanisms, whereas miR-138-5p (Bai et al., 2019) and miR-582-5p (Wang et al., 2017) directly target FOXC1. These studies primarily describe post-transcriptional regulation. The present work adds a transcriptional positive-feedback circuit between FOXC1 and β-catenin, thereby broadening the regulatory framework of FOXC1 in cancer.

AI-assisted drug discovery can accelerate early-stage molecular generation and prioritization (Ledford, 2025; Ren et al., 2025; The Lancet, 2025). In the present study, MolProphet was used to identify a predicted FOXC1 pocket and generate candidate molecules, after which FOXC1-I4 was selected for experimental characterization. Docking and molecular dynamics analyses suggested a stable interaction within the predicted FOXC1 pocket, and direct binding was supported by SPR and MST. The reduction in FOXC1-dependent reporter activity further supports FOXC1-I4 as a lead compound for subsequent structure-activity and pharmacological optimization. FOXC1-I4 is a *de novo* AI-designed inhibitor with a unique chemical skeleton.

This study has several limitations. Pharmacokinetics, metabolic stability, plasma protein binding, tissue exposure, and formal acute or repeated-dose toxicity were not evaluated. FOXC1-I4 treatment began 24 h after tumor-cell inoculation; thus, the animal experiment primarily assessed early pulmonary seeding, colonization, and subsequent outgrowth rather than treatment of established metastases. The repeated intravenous regimen was selected for experimental exposure and is not an optimized clinical route. The nude-mouse model also lacks an intact immune system. Future studies should include full analytical characterization, ADME and pharmacokinetic/pharmacodynamic evaluation, formal toxicology, imaging-confirmed established-metastasis models, clinically practical formulations and routes, and immunocompetent models.

In conclusion, the data identify a FOXC1/β-catenin positive-feedback loop that contributes to lung cancer metastasis and show that FOXC1-I4 binds FOXC1, suppresses FOXC1-dependent transcriptional activity, and reduces migration, invasion, and early pulmonary colonization in the experimental systems used. FOXC1-I4 should therefore be regarded as a lead compound that requires further chemical, pharmacokinetic, safety, and therapeutic validation rather than as a clinically established drug candidate.

## Data Availability

The original contributions presented in the study are included in the article/Supplementary Material, further inquiries can be directed to the corresponding authors.

## Supplementary material

SUPPLEMENTARY FIGURE S1
**(A)** Relative viability of A549 cells treated with FOXC1-I4 at 0, 2, 4, or 8 μM. **(B)** qRT-PCR and Western blot analysis showing that β-catenin overexpression did not significantly alter TCF4 mRNA or protein abundance. **(C)** Liquid chromatography-mass spectrometry characterization of FOXC1-I4 supplied by SanOmics. The upper panel shows the chromatogram recorded at 254 nm, and the lower panel shows the corresponding positive-ion mass spectrum with a major ion at m/z 369.9. ns, not significant.
