# Supplementary material for: FOXC1 drives lung cancer metastasis and functions as a target for AI-enabled anti-metastatic drug discovery
Source: Front Pharmacol. 2026 Sep 10;17:1868681. doi: 10.3389/fphar.2026.1868681 (PMC13601247; doi:10.3389/fphar.2026.1868681)
Supplement: Supplementary file 1 [file Supplementaryfile1.docx]

Supplementary Material

**1.Original Western blot Data.**
